# Outcome Prediction in Unresponsive Wakefulness Syndrome and Minimally Conscious State by Non-linear Dynamic Analysis of the EEG

**DOI:** 10.3389/fneur.2021.510424

**Published:** 2021-02-22

**Authors:** Baohu Liu, Xu Zhang, Lijia Wang, Yuanyuan Li, Jun Hou, Guoping Duan, Tongtong Guo, Dongyu Wu

**Affiliations:** ^1^Department of Rehabilitation, Wangjing Hospital, China Academy of Chinese Medical Sciences, Beijing, China; ^2^Department of Statistics and Actuarial Science, University of Waterloo, Waterloo, ON, Canada; ^3^Tianjin University of Traditional Chinese Medicine, Tianjin, China

**Keywords:** electroencephalogram, minimally conscious state, non-linear dynamics, prognosis, unresponsive wakefulness syndrome

## Abstract

**Objectives:** This study aimed to investigate the role of non-linear dynamic analysis (NDA) of the electroencephalogram (EEG) in predicting patient outcome in unresponsive wakefulness syndrome (UWS) and minimally conscious state (MCS).

**Methods:** This was a prospective longitudinal cohort study. A total of 98 and 64 UWS and MCS cases, respectively, were assessed. During admission, EEGs were acquired under eyes-closed and pain stimulation conditions. EEG nonlinear indices, including approximate entropy (ApEn) and cross-ApEn, were calculated. The modified Glasgow Outcome Scale (mGOS) was employed to assess functional prognosis 1 year following brain injury.

**Results:** The mGOS scores were improved in 25 (26%) patients with UWS and 42 (66%) with MCS. Under the painful stimulation condition, both non-linear indices were lower in patients with UWS than in those with MCS. The frontal region, periphery of the primary sensory area (S1), and forebrain structure might be the key points modulating disorders of consciousness. The affected local cortical networks connected to S1 and unaffected distant cortical networks connecting S1 to the prefrontal area played important roles in mGOS score improvement.

**Conclusions:** NDA provides an objective assessment of cortical excitability and interconnections of residual cortical functional islands. The impaired interconnection of the residual cortical functional island meant a poorer prognosis. The activation in the affected periphery of the S1 and the increase in the interconnection of affected local cortical areas around the S1 and unaffected S1 to the prefrontal and temporal areas meant a relatively favorable prognosis.

## Introduction

Clinically, predicting the outcome in terms of recovery from disorders of consciousness (DOC) in patients who survive coma after acute brain injury is challenging. Meanwhile, predicting patient outcome in unresponsive wakefulness syndrome (UWS) and minimally conscious state (MCS) is of great importance because it directly affects the follow-up treatment and financial burden on the patient's family and society.

Previous evidence demonstrates that MCS or UWS cases may retain islands of intact cognitive, sensory, and auditory functions (1–7). Brain regions controlling pain are distributed in a comparable network in control and MCS cases but show a wider distribution than their counterparts in UWS cases (8). In comparison with reduced cerebral activation detected in UWS cases, MCS cases show activation levels comparable to control patients upon stimulation by auditory, emotional and noxious triggers (9).

It was reported that MCS cases commonly exhibit event-related potential (ERP) components, suggesting complex information processing in the association cortex; such components are also abundant in UWS cases (10). This was confirmed by multiple research groups assessing UWS and MCS (11–13). Several studies have confirmed that N400 can predict a favorable outcome and can help evaluate the long-term prognosis of unresponsive patients with UWS and MCS through the assessment of residual cognition (14, 15).

Electroencephalogram (EEG) represents a simple, bedside, and widely used tool for predicting outcome in patients with DOC, but its value is limited by the lack of a uniform classification (16). EEG's complexity and reduced predictability result from its ultrahigh dimensionality, which makes this tool a random system. Entropy is related to the amount of “disorder” in the system. Approximate entropy (ApEn) can quantify the irregularity of data time series, that is, the predictability of subsequent amplitude values based upon the knowledge of the previous amplitude values. Several previous studies based on non-linear dynamic analysis (NDA) showed that patients with UWS having the lowest ApEn values either die or survive with UWS, while those showing the most elevated ApEn values develop MCS or partially or fully recover, suggesting that dynamic correlates of neural residual complexity might help predict the outcomes in vegetative patients (17). Patients with UWS show the lowest non-linear indices, followed by individuals with MCS. UWS and MCS cases show decreased responses to auditory and pain stimuli compared with healthy individuals (18). Cross-approximate entropy (C-ApEn) may be interpreted as a measure of the number of states independently accessible by two cortical areas. Thus, an elevation in C-ApEn during pain stimulation may indicate an increase in intercortical communication. C-ApEn measures interconnections of residual cortical functional islands with associative cortices in individuals with unconsciousness; the interconnection of local and distant cortical networks is more pronounced in MCS than in UWS (19).

However, whether UWS and MCS cases with altered interconnections of residual cortical functional islands have worse outcomes remains unclear, as does the prognostic difference between the interconnections of local and distant cortical networks in MCS and UWS.

The objective of this study was to investigate the role of NDA in outcome prediction for patients with UWS and MCS. We hypothesized that [1] impaired interconnections of residual cortical functional islands play an important role in the poor prognosis of patients with UWS and MCS; and [2] ApEn and C-ApEn can provide information on cortical excitability and the interconnections of cortical networks for predicting recovery from DOC. To explore these points, ApEn and C-ApEn were measured in all patients with UWS and MCS.

## Materials and Methods

### Participants

This trial was carried out in the Department of Rehabilitation, Xuanwu Hospital of Capital Medical University, and the Department of Rehabilitation, Wangjing Hospital, China Academy of Chinese Medical Sciences (Beijing, China), with approval from the respective ethics committees. All patient guardians or parents provided signed informed consent.

The inclusion criteria were as follows: [1] UWS or MCS diagnosed according to the Multi-Society Task Force Report on PVS (Medical aspects of the persistent vegetative state [1]. The Multi-Society Task Force on PVS (20)) and Giacino on MCS (21); [2] onset of brain injury 2–6 months before inclusion in the study; and [3] no previous brain injury. The exclusion criteria were as follows: [1] unstable vital signs; [2] overt communicating hydrocephalus; [3] diagnosis of locked-in syndrome; [4] diagnosis of primary brain-stem injury; and [5] severe spasticity, resulting in electromyography (EMG) artifacts.

This study included 162 individuals with unconsciousness following serious brain injury or stroke, comprising 98 UWS and 64 MCS cases. There were 116 and 46 male and female patients, respectively, with ages ranging from 17 to 73 years.

Medications and conventional physical therapy remained unchanged throughout hospitalization.

### Clinical Assessment

Brain magnetic resonance imaging (MRI) or computed tomography (CT) was performed for all individuals to properly locate brain injuries and to rule out communicating hydrocephalus. The Coma Recovery Scale-Revised (CRS-R) was assessed independently by two experienced raters at admission. A patient's diagnosis was based on the highest score obtained over five to seven CRS-R assessments during the day.

Modified Glasgow Outcome Scale (mGOS) scores were determined to assess functional prognosis in participants 1 year following cerebral damage. A specific category for MCS was added to the traditional GOS, with designations such as death, vegetative state (VS), MCS, severe disability, moderate disability and good recovery (22, 23). Improvement was defined as an upgrade in mGOS score; otherwise, no improvement was considered.

The patients who were discharged from the hospital 12 months earlier were investigated via telephone calls.

### EEG Recording

EEGs were recorded with the patent awake, lying comfortably in a quiet environment, using a Wireless Digital EEG (ZN16E, China). Sixteen channels were used based on the 10–20 system, and the earlobe electrode served as a reference. Signals underwent digitization with 500 Hz as the sample rate and 0.3–100 Hz as the bandwidth.

**Figure 1:**
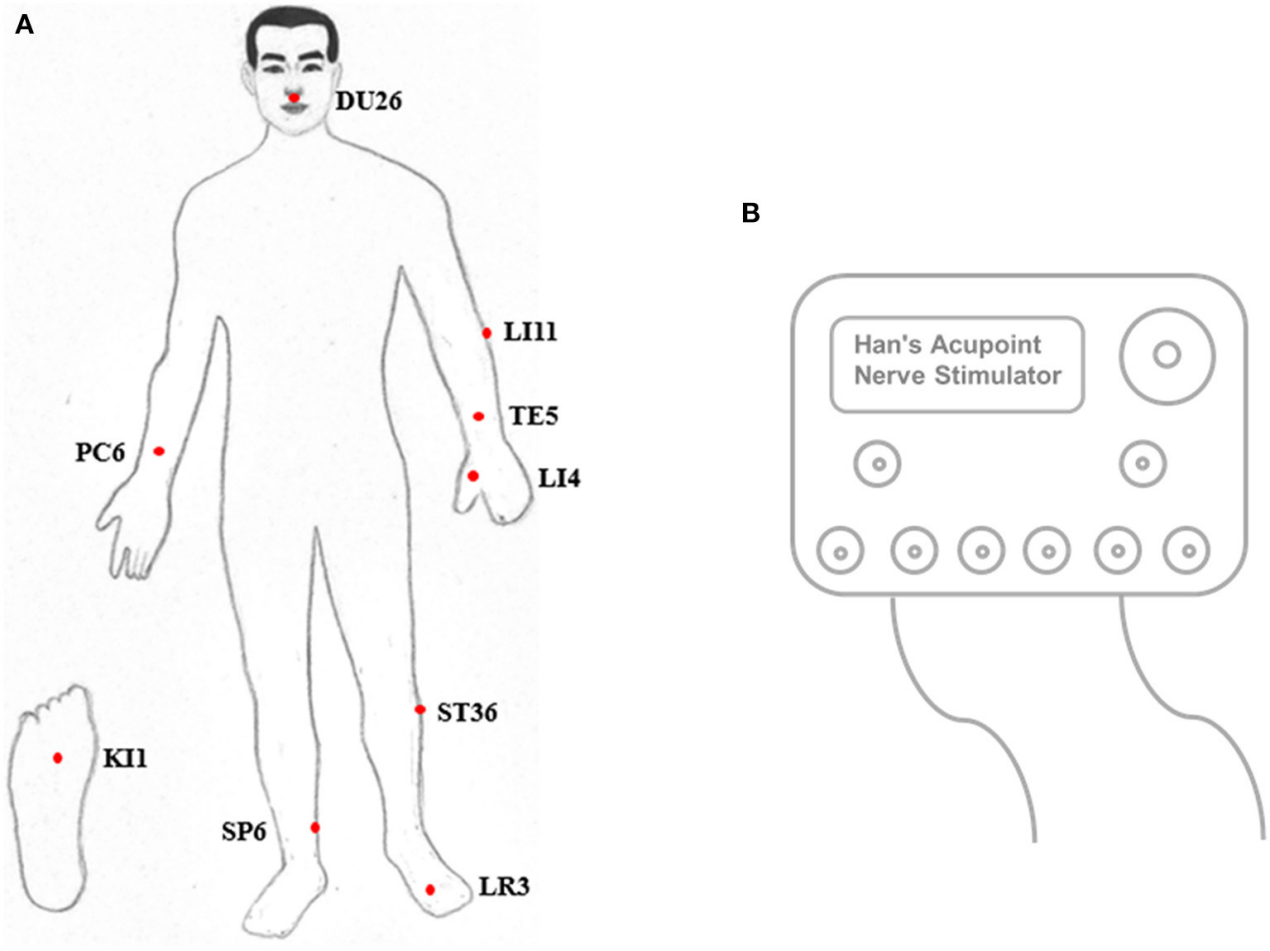
Positions of acupoints **(A)** and the Han acupoint nerve stimulator (HANS) **(B)**.

EEGs were first acquired under eyes-closed conditions for ~5 min. Next, pain was stimulated in both legs, starting with the diseased side. The acupuncture points Shuigou (DU26), Quchi (LI11), Waiguan (TE5), Neiguan (PC6), Hegu (LI4), Zusanli (ST36), Yongquan (KI1), Sanyinjiao (SP6), and Taichong (LR3) were triggered with a Han acupoint nerve stimulator (HANS) (Figure 1), during which time the EEG was recorded for ~5 min. EEG acquisition was performed as previously described (18, 19).

An artifact-free epoch was selected offline by a senior physician. A 50-Hz notch filter was employed for electrical noise removal. The subscripts of EEG montages were modified (from the left or right side to the affected or unaffected side): FP_A_, FP_U_, F_A_, F_U_, C_A_, C_U_, P_A_, P_U_, O_A_, O_U_, AT_A_ (anterior temporal), AT_U_, MT_A_ (middle temporal), MT_U_, PT_A_ (posterior temporal), and PT_U_. The affected side was determined as the more seriously impaired side through MRI or CT. If both hemispheres were damaged equally in MRI or CT imaging, the affected side was determined by the EEG result, that is, the lower average ApEn value meant more serious impairment.

### Non-linear Dynamic Analysis

#### Approximate Entropy

ApEn confers a non-negative number to a time series; the greater the value is, the higher the data complexity/irregularity. Therefore, elevated irregularity (high ApEn) reflects high complexity, i.e., elevated non-linear cell dynamics or enhanced interactions of cortical networks. The detailed algorithm has been described (18).

#### Cross-Approximate Entropy

C-ApEn analyzes two related time series to assess their asynchrony. It is very similar to ApEn in design and intent, differing only in that it compares sequences from one series with those of the second (19). The detailed algorithm has been described previously (19).

#### Data Analysis

A total of 32,768 artifact-free consecutive data points (65.536 s) were selected for the NDA. C_A_ and C_U_ with other EEG sites were calculated to determine the cortical response to pain stimuli in patients with UWS and MCS. Local and distant C-ApEn values were calculated to assess whether changes in C-ApEn were associated with impaired transmission of information over short or long distances. The mean C-ApEn values around electrodes located over the central region were estimated as the local C-ApEn, that is, C_A_-P_A_, C_A_-F_A_, C_A_-MT_A_, C_U_-P_U_, C_U_-F_U_, and C_U_-MT_U_. The distant C-ApEn pairs included C_A_-FP_A_, C_A_-O_A_, C_U_-FP_U_, and C_U_-O_U_.

### Statistical Analysis

R v3.4.2 was used to perform all analyses. Independent-sample *t*-tests were carried out to compare interval baseline variables, baseline ApEn and C-ApEn under eyes-closed conditions, and differences in ApEn and C-ApEn between the pain stimulation state and eyes-closed conditions for both groups. Multivariate *t*-tests (Hotelling t-squared tests) were also implemented to correct for the multiple comparisons between groups. Linear regression models were implemented to investigate how well EEG predicted recovery of consciousness in patients. The regression analysis was divided into two parts based on ApEn and C-ApEn analyses. ApEn, C-ApEn, and patient (age, sex, diagnosis, and duration) features were considered independent variables, and mGOS improvement was considered a dependent variable to construct the regression model. In the ApEn regression model, EEG signals from the affected side included FP_A_, F_A_, C_A_, P_A_, O_A_, AT_A_, MT_A_, and PT_A_, while those from the unaffected side were FP_U_, F_U_, C_U_, P_U_, O_U_, AT_U_, MT_U_, and PT_U_. Similarly, in the C-ApEn regression model, EEG signals from the affected side included C_A_-F_A_, C_A_-P_A_, C_A_-MT_A_, C_A_-FP_A_, and C_A_-O_A_, while those from the unaffected side were C_U_-F_U_, C_U_-P_U_, C_U_-MT_U_, C_U_-FP_U_, and C_U_-O_U_. *P* <0.05 in all analyses was considered statistically significant.

## Results

Table 1 summarizes the demographic features of the subjects. Age, sex, and disease course were comparable in both groups. In terms of diagnosis, the UWS group consisted of more stroke patients, while the MCS group had more cases of traumatic brain injury.

**Table 1:** Demographic characteristics of all patients.

**Variables**	**UWS (*n* = 98)**	**MCS (*n* = 64)**	***p***
Age (years)^a^	46.2 ± 13.7	45.5 ± 15.6	0.621
Duration (days)^a^	144.0 ± 90.1	147.6 ± 92.7	0.404
Sex^b^			
Male	66 (67.3)	50 (78.1)	
Female	32 (32.7)	14 (21.9)	0.137
Diagnosis^b^			
Traumatic brain injury	44 (44.9)	39 (60.9)	
Stroke	54 (55.1)	25 (39.1)	0.046^**^
mGOS^c^ (12 month after brain injury)	2 (2–4)	4 (3–6)	

a *Values in cells are mean ± standard deviation*.

b *Values in cells are frequency (percentage)*.

c *Values in cells are median (range)*.

** *P <0.05*.

### Patient Outcomes

Of the 98 UWS cases, 13 had severe disability (13%), 12 presented MCS (12%), 72 had VS (73%), and 1 died (1%). Of the 64 MCS cases, 2 had good recovery (3%), 5 presented moderate disability (8%), 35 had severe disability (55%), and 22 had MCS (34%). During the follow-up period, one patient with VS/UWS died within 12 months after brain injury due to pulmonary infection. Collectively, at the study endpoint, the mGOS scores were improved in 25 (26%) patients with UWS and 42 (66%) with MCS.

### Non-linear Dynamic Analysis

Table 2 presents comparisons of baseline ApEn and C-ApEn indices for both groups under eyes-closed conditions. Both parameters showed significant elevations in MCS cases in comparison with UWS patients. The results from the multivariate *t*-test (Hotelling Test) indicated that there were significant differences when performing the multiple comparisons for both ApEn and C-ApEn indices between MCS and UWS (Table 3). Tables 4, 5 list the differences in ApEn and C-ApEn between the pain stimulation and eyes-closed conditions in both groups. The ApEn results showed no significant difference between the two groups under affected pain stimulation conditions, while the values were significantly higher in the MCS group than in the UWS group under unaffected pain stimulation conditions. Both local and distant C-ApEn indices were significantly higher in the MCS group than in the UWS group under affected and unaffected pain stimulation conditions.

**Table 2:** ApEn and C-ApEn indices under eyes-closed conditions.

	**Montages**	**UWS**	**MCS**	***P***
ApEn	FP_A_	0.55 ± 0.09	0.65 ± 0.10	<0.001^***^
	FP_U_	0.55 ± 0.10	0.66 ± 0.11	<0.001^***^
	F_A_	0.58 ± 0.09	0.64 ± 0.12	<0.001^***^
	F_U_	0.56 ± 0.09	0.63 ± 0.12	<0.001^***^
	C_A_	0.60 ± 0.08	0.67 ± 0.11	<0.001^***^
	C_U_	0.60 ± 0.11	0.66 ± 0.10	<0.001^***^
	P_A_	0.60 ± 0.08	0.68 ± 0.12	<0.001^***^
	P_U_	0.60 ± 0.10	0.66 ± 0.09	<0.001^***^
	O_A_	0.61 ± 0.11	0.71 ± 0.13	<0.001^***^
	O_U_	0.62 ± 0.12	0.70 ± 0.12	<0.001^***^
	AT_A_	0.61 ± 0.11	0.69 ± 0.14	<0.001^***^
	AT_U_	0.59 ± 0.13	0.71 ± 0.11	<0.001^***^
	MT_A_	0.66 ± 0.13	0.72 ± 0.13	<0.001^***^
	MT_U_	0.64 ± 0.15	0.74 ± 0.12	<0.001^***^
	PT_A_	0.66 ± 0.13	0.75 ± 0.14	<0.001^***^
	PT_U_	0.65 ± 0.13	0.71 ± 0.11	<0.001^***^
C-ApEn	C_A_- F_A_	0.73 ± 0.09	0.82 ± 0.13	<0.001^***^
	C_A_- P_A_	0.73 ± 0.09	0.84 ± 0.14	<0.001^***^
	C_A_- MT_A_	0.79 ± 0.09	0.88 ± 0.14	<0.001^***^
	C_A_- FP_A_	0.74 ± 0.09	0.85 ± 0.12	<0.001^***^
	C_A_- O_A_	0.77 ± 0.09	0.88 ± 0.14	<0.001^***^
	C_U_- F_U_	0.72 ± 0.11	0.80 ± 0.13	<0.001^***^
	C_U_- P_U_	0.73 ± 0.10	0.81 ± 0.14	<0.001^***^
	C_U_-MT_U_	0.78 ± 0.12	0.86 ± 0.13	<0.001^***^
	C_U_-FP_U_	0.75 ± 0.11	0.83 ± 0.12	<0.001^***^
	C_U_- O_U_	0.77 ± 0.11	0.87 ± 0.14	<0.001^***^

*Values in cells are mean ± standard deviation*.

*** *P < 0.01*.

**Table 3:** Multivariate *t*-tests for differences between the painful stimulation condition and the eyes-closed condition for both groups.

	**Condition**	**Test statistics**	***P***
ApEn	Affected side	1.639	0.0657^*^
	Unaffected side	2.158	0.008816^***^
C-ApEn	Affected side	6.842	<0.001^***^
	Unaffected side	10.621	<0.001^***^

Significant codes:

* P < 0.1.

*** *P < 0.01*.

**Table 4:** ApEn differences between the painful stimulation condition and the eyes-closed condition for both groups.

**Condition**	**Montages**	**UWS**	**MCS**	***P***
Affected side	FP_A_	−0.04 ± 0.09	−0.03 ± 0.11	0.231
	F_A_	−0.03 ± 0.06	−0.04 ± 0.07	0.785
	C_A_	−0.03 ± 0.06	−0.03 ± 0.11	0.623
	P_A_	−0.03 ± 0.06	−0.02 ± 0.07	0.209
	O_A_	−0.02 ± 0.09	−0.00 ± 0.10	0.139
	AT_A_	−0.04 ± 0.09	−0.03 ± 0.09	0.458
	MT_A_	−0.02 ± 0.11	−0.01 ± 0.09	0.311
	PT_A_	−0.02 ± 0.10	−0.00 ± 0.11	0.149
Unaffected side	FP_U_	0.05 ± 0.09	0.09 ± 0.12	0.004^***^
	F_U_	0.04 ± 0.06	0.09 ± 0.08	<0.001^***^
	C_U_	0.04 ± 0.06	0.08 ± 0.09	0.002^***^
	P_U_	0.04 ± 0.06	0.10 ± 0.09	<0.001^***^
	O_U_	0.06 ± 0.10	0.11 ± 0.11	<0.001^***^
	AT_U_	0.07 ± 0.12	0.11 ± 0.11	0.030^**^
	MT_U_	0.09 ± 0.13	0.11 ± 0.09	0.072^*^
	PT_U_	0.08 ± 0.13	0.12 ± 0.12	0.025^**^

*Values in cells are mean ± standard deviation*.

* P < 0.1.

** P < 0.05.

*** *P < 0.01*.

**Table 5:** C-ApEn differences between the painful stimulation condition and the eyes-closed condition for both groups.

**Condition**	**Montages**	**UWS**	**MCS**	***P***
Affected side	C_A_- F_A_	−0.03 ± 0.06	−0.01 ± 0.07	0.021^**^
	C_A_- P_A_	−0.02 ± 0.05	−0.00 ± 0.08	0.025^**^
	C_A_- MT_A_	−0.04 ± 0.06	−0.00 ± 0.08	<0.001^***^
	C_A_- FP_A_	−0.03 ± 0.06	0.01 ± 0.10	0.004^***^
	C_A_- O_A_	−0.03 ± 0.07	−0.01 ± 0.06	0.020^**^
Unaffected side	C_U_- F_U_	0.02 ± 0.06	0.09 ± 0.07	<0.001^***^
	C_U_- P_U_	0.02 ± 0.06	0.10 ± 0.08	<0.001^***^
	C_U_-MT_U_	0.02 ± 0.07	0.11 ± 0.08	<0.001^***^
	C_U_-FP_U_	0.02 ± 0.06	0.11 ± 0.09	<0.001^***^

*Values in cells are mean ± standard deviation*.

* P < 0.1.

** P < 0.05.

*** *P < 0.01*.

### Regression Analysis of ApEn and C-ApEn

Age, sex, diagnosis, and duration were not the main relevant factors determining mGOS improvement.

For ApEn, Table 6 shows that the CRS-R score, P_A_, and O_A_ were the main factors associated with mGOS improvement under affected pain stimulation conditions, while the CRS-R score, F_U_, and C_U_ were the main factors under unaffected pain stimulation conditions.

**Table 6:** Regression analysis of ApEn.

**Affected side**					**Unaffected side**				
**Coefficients**	**Estimate**	**Std. error**	***t***	**Pr(>|t|)**	**Coefficients**	**Estimate**	**Std. error**	***t***	**Pr(>|t|)**
Intercept	−0.5320	0.6460	−0.824	0.4114	Intercept	0.5345	0.6074	−0.88	0.3803
Sex	0.0142	0.1103	0.129	0.8974	Sex	0.0471	0.1075	0.439	0.6616
Age	−0.0025	0.0036	−0.686	0.4937	Age	0.0002	0.0033	0.066	0.9471
Duration	−0.0002	0.0005	−0.461	0.6452	Duration	−0.0005	0.0005	−0.983	0.3272
TBI	0.1022	0.5930	0.172	0.8634	TBI	−0.0088	−0.5445	−0.016	0.9871
Stroke	0.0771	0.5856	0.132	0.8955	Stroke	−0.0759	0.5388	−0.141	0.8882
CRS-R	0.1068	0.0147	7.25	2.19E-11^***^	CRS-R	0.0802	0.0146	5.51	1.57E-07^***^
FP_A_	0.0771	0.5520	0.14	0.8891	FP_U_	−0.8664	0.4945	−1.752	0.0819^*^
F_A_	−0.9482	1.1007	−0.861	0.3904	F_U_	2.3388	0.9976	2.344	0.0204^**^
C_A_	−0.4503	0.6744	−0.668	0.5054	C_U_	2.1104	0.9221	2.289	0.0235^**^
P_A_	2.0450	0.9861	2.074	0.0398^**^	P_U_	0.6965	0.9018	0.772	0.4412
O_A_	−1.2194	0.5907	−2.064	0.0407^**^	O_U_	−0.3663	0.5253	−0.697	0.4867
AT_A_	1.0590	0.7487	1.415	0.1593	AT_U_	−0.4571	0.6294	−0.726	0.4688
MT_A_	0.2478	0.5828	0.425	0.6713	MT_U_	0.4794	0.5492	0.873	0.3841
PT_A_	0.5476	0.5751	0.952	0.3425	PT_U_	0.3569	0.4711	0.758	0.4498
Residual standard error: 0.5713 on 147 degrees of freedom					Residual standard error: 0.5202 on 147 degrees of freedom				
Multiple R-squared: 0.3886, Adjusted R-squared: 0.3304					Multiple R-squared: 0.4931, Adjusted R-squared: 0.4449				
F-statistic: 6.674 on 14 and 147 DF, p-value: 2.239e-10					F-statistic: 10.22 on 14 and 147 DF, p-value: 9.225e-16				

Significant codes:

* P < 0.1.

** P < 0.05.

*** *P < 0.01*.

For C-ApEn, Table 7 shows that the CRS-R score, C_A_-P_A_, and C_A_-MT_A_ were the main factors associated with mGOS improvement under affected pain stimulation conditions, while C_U_-MT_U_ and C_U_-FP_U_ were the main factors under unaffected pain stimulation conditions.

**Table 7:** Regression analysis of C-ApEn.

**Affected side**					**Unaffected side**				
**Coefficients**	**Estimate**	**Std. error**	***t***	**Pr(>|t|)**	**Coefficients**	**Estimate**	**Std. error**	***t***	**Pr(>|t|)**
Intercept	−0.0567	0.6038	−0.094	0.9252	Intercept	0.5012	0.5085	0.986	0.3259
Sex	0.0311	0.1016	0.306	0.7600	Sex	−0.0434	0.0874	−0.497	0.6201
Age	−0.0015	0.0032	−0.475	0.6353	Age	−0.0015	0.0027	−0.573	0.5678
Duration	−0.0005	0.0005	−1.029	0.3051	Duration	−0.0003	0.0004	−0.881	0.3795
TBI	−0.1640	0.5477	−0.299	0.7651	TBI	−0.2957	0.4652	−0.636	0.5259
Stroke	−0.2275	0.5417	−0.42	0.6751	Stroke	−0.4104	0.4595	−0.893	0.3731
CRS-R	0.0905	0.0143	6.316	2.89E-09^***^	CRS-R	0.0133	0.0151	0.883	0.3785
C_A_-F_A_	−1.7007	1.1568	−1.47	0.1436	C_U_-F_U_	1.2919	1.1992	1.077	0.2831
C_A_-P_A_	3.2025	1.1056	2.897	0.00434^***^	C_U_-P_U_	0.3015	1.1039	0.273	0.7851
C_A_-MT_A_	1.8856	0.8917	2.115	0.03612^**^	C_U_-MT_U_	2.3110	0.7873	2.935	0.0039^***^
C_A_-FP_A_	−1.2958	0.7771	−1.667	0.09752^*^	C_U_-FP_U_	2.1806	0.9376	2.326	0.0214^**^
C_A_-O_A_	1.7777	0.9286	1.922	0.05495^*^	C_U_-O_U_	0.6155	0.8426	0.73	0.4663
–	–								
Residual standard error: 0.5262 on 150 degrees of freedom					Residual standard error: 0.4452 on 150 degrees of freedom				
Multiple R-squared: 0.4708, Adjusted R-squared: 0.432					Multiple R-squared: 0.6212, Adjusted R-squared: 0.5934				
F-statistic: 12.13 on 11 and 150 DF, p-value: 4.136e-16					F-statistic: 22.36 on 11 and 150 DF, p-value: <2.2e-16				

Significant codes:

* P < 0.1.

** P < 0.05.

*** *P < 0.01*.

## Discussion

This study first demonstrated that impaired interconnections of residual cortical functional islands might be useful in predicting the outcome of consciousness in patients with MCS and UWS. The results confirmed both proposed hypotheses. First, C-ApEn could assess interconnections of residual cortical functional islands, and significant differences in the number of cortical residual functional islands were found between the UWS and MCS groups. Both the local and distant C-ApEn indices were significantly higher in the MCS group than in the UWS group under pain stimulation conditions, suggesting that interconnections of residual cortical functional islands show more severe impairment in the UWS group than in the MCS group. Moreover, the mGOS improvement levels in the UWS and MCS groups were 26 and 66%, respectively, suggesting that altered interconnections of residual cortical functional islands reflect poor prognosis. Second, regression analysis showed that cortical excitability around the primary sensory area (S1) (P_A_, F_U_, and C_U_) under pain stimulation conditions (ApEn) and the local interconnection of S1 with parietal and middle temporal areas on the affected side (C_A_-P_A_ and C_A_-MT_A_) along with local and distant interconnections of S1 with the frontal area on the unaffected side (C_U_-MT_U_ and C_U_-FP_U_) reflect a more favorable outcome.

### Status Quo of the Prognostic Assessment of DOC

#### EEG

Applied to DOC, EEG focuses on the objective assessment of EEG signals and aims to detect subtleties that may escape visual inspection, thus minimizing subjectivity and human error in prognostication (24–26). The majority of cases in VS have a seriously generalized slowing of baseline activity, with delta (δ) rhythms not reacting to stimulation; cases with the severest form of VS show electrocerebral silence (27). Most patients with MCS show lower-frequency oscillations (i.e., delta, theta, and alpha bands) (28, 29). Some EEG parameters are associated with poor outcome and could eventually help predict survival. Johnsen et al. used EEG reactivity to evaluate 39 comatose patients. The results suggested that an increase in EEG activity predicts poor outcome with high specificity and modest sensitivity, and pain was the most provocative stimulation followed by sound and eye opening (30). However, individual classifications were not appropriately assessed for their predictive values (31, 32).

Resting-state EEG recording and analysis have been recently applied to DOC. The prognostic value of resting-state EEG in predicting survival or non-survival 6 months after brain injury was also proven by EEG oscillatory microstate analyses (28). Chennu and colleagues examined 104 patients with DOC at rest with high-density EEG and found that connectivity hubs in specific frontal and parietal loci play an important role in the recovery of consciousness after brain injury (33). Using resting-state EEG, Cacciola et al. investigated the functional connectivity of UWS and MCS patients from a topological network perspective, and the results confirmed that activation of the frontoparietal network is closely related to the restoration of consciousness (34). Taken together, both Chennu and Cacciola highlighted that brain networks can predict the diagnosis and prognosis of DOC at the bedside. In particular, the conclusion that the activation of the frontoparietal network is closely related to the restoration of consciousness is consistent with our study.

#### ERP

A meta-analysis suggested that MMN and P300 predict recovery in individuals showing low responses after stroke, brain trauma, and metabolic encephalopathy, respectively (35). P300 has prognostic potential in patients with DOC who have higher-order cortical information processing (36). MMN shows high specificity as a predictor of recovery of consciousness (37). Steppacher and collaborators believed that N400 is an important tool to assess information-processing capacities that could predict the likelihood of recovery in patients with UWS or MCS (15). Passive paradigms could help detect residual brain activity in patients with severe brain injury; however, the use of active paradigms (i.e., tasks requiring the patient's participation instead of passive listening) helps determine whether the patient is conscious (38).

However, these reports mainly focused on the acute phase of brain injury; studies on patients with chronic DOC are lacking. In addition, patients with DOC often have different degrees of aphasia, cognitive impairment, and/or neurobehavioral disorders (e.g., apathy), whose severities also affect the accuracy of active paradigms.

#### fMRI and PET

The prevalence of preserved consciousness in VS and MCS cases as revealed by fMRI and EEG was meta-analyzed, and active paradigms were found to likely underestimate the consciousness level in comparison with passive paradigms (39). Perhaps most notably, the strength of functional connectivity within the default mode network, as determined by resting-state fMRI, was shown to linearly correlate with the level of consciousness after traumatic coma, as determined by the CRS-R (40). On the whole, an important advantage of the resting-state fMRI technique is its ability to analyze multiple resting-state networks within a single dataset; however, its clinical application is not widespread due to the lack of related studies (41).

Previous studies performed PET to demonstrate that pain stimulation in patients with MCS leads to activation in the contralateral thalamus and S1, as in patients with PVS (8). Stender and colleagues examined 131 patients with DOC and found that FDG-PET thus constitutes a strong diagnostic and prognostic marker in DOC arising from brain injury, irrespective of pathogenesis (42).

A variety of unique challenges are inherent to applying advanced neuroimaging techniques to individuals diagnosed with DOC. First, the patient population is prone to medical instability, making it difficult to acquire functional imaging data safely. One limitation specific to PET is that it necessitates intravenous injection of a radioactive tracer, although the exposure to harmful radiation is minimal (43).

A systematic review and meta-analysis was performed to compare the prognostic effects of neurophysiological methods (EEG, ERP, fMRI, and PET). The oscillatory EEG responses were the best, and the poorest prognostic effects were shown for fMRI responses to stimulation and the ERP component P300 (44).

#### TMS-EEG

Recently, TMS-EEG was proposed to evaluate the consciousness state in patients with severe brain injury and impaired communication (45). Ragazzoni and collaborators found that TMS-EEG is a promising approach in detecting and tracking the recovery of consciousness in patients with prolonged DOC (46). Another study demonstrated that TMS-EEG might be an efficient assessment tool for evaluating the therapeutic efficiency of the rTMS protocol in DOC (47).

The limitations of this work should be mentioned. First, the study population was relatively small. A single stimulation site was used, and the M1 region is easily affected by brain damage. In addition, TMS-EEG has technical limitations, e.g., amplifiers require special functions to overcome the artifacts of the magnetic field generated by TMS in EEG (48).

### Why Was Pain Stimulation Used?

A pain stimulation protocol was designed to provide better methodological rigor in this study. Li and colleagues (22) found that thermal stimulation–induced EEG-R shows good predictive accuracy and can discriminate between patients with improved outcomes and those without. This study provided a valuable method for outcome prediction in patients with UWS or MCS. Although painful and thermal sensations share the same afferent pathways, pain stimulation could be easier to operate. Moreover, pain stimulation causes more intensive and extensive cortical activation than thermal stimulation.

Acupuncture was performed for pain stimulation. Acupuncture, widely applied in traditional Chinese medicine, and its Western derivative, electroacupuncture, have been used for treating chronic pain syndrome, as proposed by the National Institutes of Health (NIH) Consensus (49). Evidence from functional MRI (fMRI) supports the view that stimulation of acupuncture points ST36, SP6, LR3, KI1, PC6, TE5, DU26, LI4, and LI11 could activate primary and secondary somatosensory areas as well as other subcortical areas, such as the insula, thalamus cerebellum, dorsolateral prefrontal cortex and parahippocampal gyrus. These acupoints could be stimulated for brain activation and consciousness restoration, and acupuncture is consequently considered a resuscitation method (50–55). Therefore, pain stimulation caused by multiple acupoints with HANS was used to reach the maximal effect of cortical activation.

### Evidence of Cortical Excitability and Interconnection

Similar to previous studies, both non-linear indices were lower in patients with UWS than in the MCS group under eyes-closed conditions in this study (18, 19). Under pain stimulation conditions, both non-linear indices were still lower in patients with UWS than in MCS patients, except for ApEn indices under affected pain stimulation conditions, which might be associated with severe impairment of the affected cortex in both groups. The affected ApEn indices failed to distinguish UWS from MCS, but affected local and distant C-ApEn indices showed differences, indicating that C-ApEn has higher sensitivity than ApEn.

Linear regression analysis revealed that ApEn, CRS-R score, P_A_ (affected parietal), and O_A_ (affected occipital) under affected pain stimulation conditions and CRS-R score, C_U_, and F_U_ under unaffected pain stimulation conditions were associated with mGOS improvement. These results indicated that activation in the affected parietal and unaffected frontal and central areas under pain stimulation conditions reflected a relatively favorable prognosis. In most patients, there were no differences in ApEn between the pain stimulation and eyes-closed conditions in O_A_, implying no cortical excitability.

C-ApEn, CRS-R score, C_A_-P_A_, and C_A_-MT_A_ under affected pain stimulation conditions were the main factors related to mGOS improvement, while mostly C_U_-MT_U_ and C_U_-FP_U_ under unaffected pain stimulation conditions showed associations with mGOS improvement. These results indicated that increased interconnections of affected local cortical areas around C_A_ and unaffected C_U_ to temporal (local) and prefrontal (distant) areas suggest a relatively favorable prognosis.

In summary, these findings revealed frontal (F_U_), peripheral S1 (C_A_, C_U_, and P_A_), and forebrain structure (FP_U_) as potential key points for modulating DOC abnormalities. Laureys et al. investigated cortico-cortical connectivity in the DOC by ^18^F-FDG-PET in a small cohort of patients with VS/UWS. The results showed impaired regional cerebral glucose metabolism in the prefrontal, premotor, and parietotemporal association areas, in addition to the posterior cingulate cortex and precuneus (56). This finding was in agreement with the present study.

In addition, the global connectivity hypothesis in DOC emphasizes the importance of multiple networks: disruption of multiple networks simultaneously leads to unconsciousness, and recovery requires simultaneous reintegration of multiple networks (43). The above results showed that affected local cortical networks connecting S1 and unaffected distant cortical networks connecting S1 to the prefrontal area played important roles in mGOS improvement. This finding verified the global connectivity hypothesis.

### Limitations

First, the follow-up in this study was relatively simple, lacking objective assessment indices (such as EEG). Second, nonlinear EEG under pain stimulation combined with high-order cortical information processing (e.g., P300) may also improve the prediction sensitivity and specificity.

## Conclusions

NDA provides an objective evaluation of cortical excitability and interconnections of residual cortical functional islands with associative cortices in patients with unconsciousness. Altered interconnection of the residual cortical functional island reflects poor prognosis. Activation in the affected periphery of S1 and increased interconnections of affected local cortical areas around S1 and from unaffected S1 to prefrontal and temporal areas indicate a relatively favorable prognosis.

## Data Availability Statement

The datasets generated for this study are available on request to the corresponding author.

## Ethics Statement

The studies involving human participants were reviewed and approved by the ethics committee of Wangjing Hospital of China Academy of Chinese Medical Sciences (WJEC-KT-2017-014-P002). The ethics committee of Xuanwu Hospital of Capital Medical University (2011-11). Written informed consent to participate in this study was provided by the participants' legal guardian/next of kin.

## Author Contributions

BL and XZ made substantial contributions to data analysis and drafting the manuscript. LW made substantial contributions to data statistics. YL, JH, GD, and TG treated the patient and acquired the data. DW designed the study, supervised the initial drafting, and critically revised the manuscript. All authors read and approved the final manuscript.

## Conflict of Interest

The authors declare that the research was conducted in the absence of any commercial or financial relationships that could be construed as a potential conflict of interest.
